# Fatty Acid Composition, at Equivalent Lipid Exposure, Dictates Human Macrophage Polarization via PPARγ Signaling

**DOI:** 10.3390/cells15030308

**Published:** 2026-02-06

**Authors:** Halemah AlSaeed, Hesah Almusallam, Shayndel Menezes, Hessah Almelaifi, Hussah Alonaizi, Mohammad Almejaimi, Rasheed Ahmad, Fatema Al-Rashed

**Affiliations:** 1 Immunology and Microbiology Department, Dasman Diabetes Institute, Al-Soor Street, P.O. Box 1180, Dasman, Kuwait City 15462, Kuwait; halemah.alsaeed@dasmaninstitute.org (H.A.); shayndelmenezes@gmail.com (S.M.); hessalonaizi@gmail.com (H.A.); dr.almejaimi@gmail.com (M.A.); 2 Animal and Imaging Core Facility, Dasman Diabetes Institute, Dasman, Kuwait City 15462, Kuwait; hessah.almusallam@dasmaninstitute.org

**Keywords:** PPARs, fatty acid, high fat diet, inflammation, macrophages

## Abstract

**Highlights:**

**What are the main findings?**

- Fatty acid composition, rather than total fat content, directs inflammatory polarization in primary human macrophages, with distinct effects on M1-like and M2-like phenotypes.
- Palmitate-enriched lipid ratios are associated with PPARγ induction and ER-stress signaling, coinciding with pro-inflammatory polarization, whereas unsaturated fat-dominant ratios preferentially enhance PPARα and IRF4 expression and favor anti-inflammatory macrophage profiles.

**What are the implications of the main findings?**

- PPARγ functions as a context-dependent lipid sensor that links fatty acid composition to macrophage inflammatory programming in human systems.
- These findings highlight the nutritional and therapeutic relevance of fatty acid composition, particularly unsaturated fat-enriched lipid formulations, in shaping innate immune responses and inflammatory tone.

**Abstract:**

Dietary fats are consumed as mixtures, yet it remains unclear whether fatty acid composition, independent of fat content, dictates human macrophage polarization. We compared two defined mixtures containing identical fatty acids (palmitic, oleic, and linoleic acids) in different ratios: a palmitate-enriched mixture (4:3:3) and an unsaturated fat-dominant mixture (2:4:4). In primary human monocyte-derived macrophages, palmitate enrichment increased CD14+CD11b+HLA-DR+ pro-inflammatory polarization, whereas the unsaturated fat-dominant mixture increased CD14+CD11b+CD163+ anti-inflammatory polarization. Mechanistic studies in THP-1-derived macrophages recapitulated these phenotype shifts and identified a reciprocal nuclear-receptor program: palmitate enrichment induced peroxisome proliferator-activated receptor gamma (PPARγ), together with ER-stress mediators EIF2AK3 and DDIT3, while the unsaturated fat-dominant mixture preferentially induced PPARα and IRF4. Pharmacologic modulation demonstrated functional dependence on PPARγ: GW9662 attenuated palmitate-driven M1-like polarization, whereas rosiglitazone disrupted the protective program under unsaturated fat-dominant conditions. These findings show that fatty acid composition, at equivalent total lipid concentration, is a dominant determinant of human macrophage inflammatory fate and highlight PPARγ as a context-dependent lipid sensor.

## 1. Introduction

Obesity is increasingly recognized as a state of chronic, low-grade inflammation characterized by metabolic stress, adipose tissue remodeling, and dysregulated lipid handling. In this inflammatory milieu, circulating monocytes and tissue macrophages play central roles in shaping metabolic homeostasis as they respond dynamically to nutrient excess, free fatty acids, lipopolysaccharide leakage, and cytokine-driven signals. Our group and others have shown that saturated fatty acids such as palmitate promote inflammatory activation, ER stress, and oxidative stress pathways through mechanisms involving ACSL1, IL-6R/gp130 signaling, and TLR4-IRF3 axes, whereas unsaturated fatty acids and omega-3 lipids such as EPA can mitigate inflammatory and oxidative responses [1,2,3]. These findings highlight the importance of dietary lipid quality, rather than quantity, in modulating macrophage phenotype and innate immune tone. Against this backdrop, the ratio of saturated to unsaturated fatty acids emerges as a physiologically relevant determinant of immune programming in obesity-associated inflammation. Macrophages are central orchestrators of innate immunity and are highly responsive to metabolic cues within their microenvironment. Among these cues, dietary fatty acids have emerged as potent modulators of macrophage polarization, influencing the balance between pro-inflammatory (M1) and anti-inflammatory (M2) phenotypes [4]. Saturated fatty acids such as palmitic acid (PA) are generally associated with inflammation and metabolic dysfunction, whereas unsaturated fatty acids like oleic acid (OA) and linoleic acid (LA) are known to exert immunomodulatory and protective effects [5,6]. However, while the individual immunological roles of these fatty acids are well-characterized, less is known about how the relative proportions of these fats, rather than their mere presence or absence, affect immune cell behavior.

In vivo, dietary fats are consumed as complex mixtures, not isolated species. Thus, the immunological outcome is likely shaped not only by the type of fatty acid but by their relative ratios. The ability of immune cells to interpret and integrate these lipid cues depends, in part, on lipid-sensing nuclear receptors, most notably the Peroxisome Proliferator-Activated Receptor (PPAR) family [7]. PPARγ, PPARα, and PPARδ regulate distinct arms of lipid metabolism, inflammatory gene expression, and macrophage differentiation. While PPARγ is classically viewed as anti-inflammatory, emerging evidence suggests that its effects are context-dependent and may vary with the lipid environment [8].

To address this knowledge gap, we designed two defined fatty acid mixtures comprising identical components—PA, OA, and LA—but differing in their relative ratios. The palmitate-enriched fatty acid mixture, termed the “high-fat diet” (HFD), was enriched in saturated fat (PA:OA:LA = 4:3:3), while unsaturated fat-dominant fatty acid mixtures, termed the “good-fat diet” (GFD), favored unsaturated fats (PA:OA:LA = 2:4:4). Using a human macrophage model, we evaluated how these lipid ratios influence cellular morphology, lipid uptake, inflammatory phenotype, and the expression of PPAR isoforms. Additionally, we explored downstream transcriptional regulators, including IRF4, STAT3, SREBF1/2, and DDIT3, to dissect the mechanistic underpinnings of diet-induced macrophage programming.

It is important to emphasize that the present study is focused on the early lipid-sensing and transcriptional events that occur within the first 24 h of macrophage exposure to defined fatty acid compositions. Acute responses such as PPAR activation, ER-stress signaling, oxidative stress responses, and early M1/M2 polarization are known to occur rapidly and shape the subsequent inflammatory trajectory. Our group and others have extensively shown that short-term fatty acid or LPS exposure (12–24 h) is sufficient to induce robust macrophage programming, including ACSL1-dependent inflammatory activation, IL-6R–driven lipid handling changes, SCFA/ACSL1-mediated cytokine induction, and EPA-mediated mitigation of LPS-induced oxidative stress and inflammation [1,2,3,9]. These studies collectively validate the physiological relevance of acute exposure models for dissecting early mechanistic events. Accordingly, the objective of the present work is to investigate these initiation mechanisms while acknowledging that longer exposures (48–72 h) are required to model chronic or unresolved inflammation. This acute timeframe was intentionally selected, as prolonged fatty acid exposure in THP-1-derived macrophages following PMA differentiation is known to compromise cell viability and confound inflammatory readouts.

This study provides new insights into how lipid composition, not just content, can fine-tune macrophage function through PPAR-dependent and PPAR-independent transcriptional networks, with implications for nutritional immunology and metabolic disease.

## 2. Materials and Methods

### 2.1. Peripheral Blood Mononuclear Cells (PBMCs)

The PBMCs used in this study were derived from human donors enrolled in a previously published study [10]. In that study, participants provided written informed consent for both the primary analyses and for potential future use of their biological samples. Ethical approval for sample collection and future research use was obtained from the Kuwait Ministry of Health Ethical Board (Approval ID: 2017/542) and the Ethical Review Committee of the Dasman Diabetes Institute, Kuwait City, KuwaitApproval ID#: RA HM-2019-019). PBMCs were isolated by density gradient centrifugation (Ficoll-Paque), cryopreserved in fetal bovine serum with 10% DMSO, and stored in liquid nitrogen until use. For the current work, only previously collected and consented cells were used. For experiments using primary human monocyte-derived macrophages, biological replicates represent cells derived from independent human donors. Unless otherwise stated, n = 3 indicates biological replicates from three different donors. Within each biological replicate, technical replication was performed as follows: quantitative PCR (TaqMan assays) was run in duplicate, while flow cytometry analyses were performed with three technical replicates per donor.

### 2.2. Cell Lines

Human THP-1 monocytes obtained from (ATCC, Cat#TIB-202, American Type Culture Collection, Manassas, VA, USA)) were seeded in 6-wells plate and maintained at 5% CO_2_ and 37 °C in RPMI-1640 medium (Gibco^TM^, Thermo Fisher Scientific, Waltham, MA, USA) containing 10% fetal bovine serum, 2 mM L-glutamine, 1 mM sodium pyruvate, 10 mM HEPES, 100 µg/mL Normocin, and 50 U/mL penicillin–streptomycin. THP-1 cells were treated with 10 ng/mL phorbol 12-myristate 13-acetate (PMA; Sigma -Aldrich, Cat# P1585, St. Louis, MO, USA) for 72 h to induce differentiation into macrophages and then cultured for an additional 72 h in PMA-free medium before experimental treatments.

### 2.3. Preparation of BSA–Fatty Acid Complexes

Bovine serum albumin (BSA) and fatty acids (FA) complexes were prepared using Harken, Dixon, and Heimber methods [11,12]. A 24% (*w*/*v*) BSA solution was prepared by gradually dissolving fatty acid-free BSA (Cat #A6003, Sigma-Aldrich, St. Louis, MO, USA) in NaCl to reach a final ration of 7.4 pH. BSA was then filtered through a 0.22 μm filter, aliquoted and stored at –20 °C until use. To prepare the fatty acid/BSA solution, oleic acid (OA) (Sigma, Cat#O1008-5G), palmitate acid (PA) (Cat#P0500-10G, Sigma-Aldrich, St. Louis, MO, USA), and linoleic acid (LA) (Cat#L1376-10MG, Sigma-Aldrich, St. Louis, MO, USA) were added individually to the 24% BSA solution to a final concentration of 10 mM for each FA. Fatty acid mixtures were gently heated and stirred on a hot plate (IKA, Staufen, Germany) until emulsions formed. The emulsions were then stored at −20 °C until needed.

### 2.4. Cell Treatment

Two mixtures of the previously prepared fatty acids were created: an HFD (PA:OA:LA = 4:3:3 ratio) and a GFD (OA:PA:LA = 2:4:4 ratio). For each well of seeded cells, 150 μM of fatty acid was added and incubated for 24 h. The 24 h exposure was chosen to capture early inflammatory and nuclear receptor-dependent signaling under non-cytotoxic conditions, as higher palmitate concentrations or prolonged exposure are known to induce apoptosis and confound interpretation of inflammatory phenotypes.

To study PPARγ activity, two drugs were administered: GW9662 (3.3 nM; M6191, Sigma-Aldrich, St. Louis, MO, USA) as a PPARγ antagonist and rosiglitazone (1 µM; 557366-M; Millipore, Burlington, MA, USA) as a PPARγ agonist. The drugs were added to cultured cells and incubated for one hour, followed by overnight incubation with HFD and GFD fatty acids.

### 2.5. Reverse Transcription Quantitative PCR (qPCR)

Total RNA was extracted from cultured macrophages using the RNeasy Mini Kit (Cat# 74104; Qiagen, Hilden, Germany) following the manufacturer’s protocol. RNA concentration and purity were assessed using a NanoDrop 2000c spectrophotometer (Thermo Fisher Scientific, Waltham, MA, USA), and 1 µg of RNA was used for complementary DNA (cDNA) synthesis with the High-Capacity cDNA Reverse Transcription Kit (Cat# 4368814; Applied Biosystems, Foster City, CA, USA).

Quantitative PCR (qPCR) was performed on a 7500 Fast Real-Time PCR System (Applied Biosystems, Foster City, CA, USA) using TaqMan^®^ Gene Expression Master Mix (Cat# 4369016; Applied Biosystems, Foster City, CA, USA) and inventoried TaqMan^®^ Gene Expression Assays. Each reaction contained 25 ng of cDNA template in a total volume of 20 µL.

For each sample, the threshold cycle (Ct) value of the target gene was normalized to the housekeeping gene GAPDH to generate a ΔCt value:

**ΔC*_t_* = C*_t_* target − C*_t_* GAPDH**

Relative expression was calculated using the ΔΔCt method, in which ΔCt values from treated samples were compared to the corresponding control group:

**ΔΔC*_t_* = ΔC*_t_ *treated − ΔC*_t_* control**

Fold change in gene expression was determined as

**Fold Change = 2 − ΔΔC*_t_***

Control samples were set to a value of 1. Data are presented as mean ± SEM from at least three biological replicates. A complete list of TaqMan assay IDs used in the study is provided in Supplementary Table S1.

### 2.6. Western Blotting

Western blotting was performed as previously described in our earlier work, using standard SDS-PAGE separation, nitrocellulose transfer, and ImageJ software (version 1.54f) (National Institutes of Health, Bethesda, MD, USA)-based densitometric quantification [1]. In brief, THP-1 cells were lysed in buffer containing protease inhibitors, and total protein concentration was determined using the Bradford assay (Bio-Rad Laboratories, Hercules, CA, USA). Equal amounts of protein were resolved on 10% SDS–PAGE gels and transferred onto nitrocellulose membranes (Bio-Rad Laboratories, Hercules, CA, USA). Membranes were blocked with Blotto A (non-fat dry milk; Cat# sc-2324; Santa Cruz Biotechnology, Dallas, TX, USA) for 1 h at room temperature and incubated overnight at 4 °C with primary antibodies. Multiplex Western blotting was performed using infrared dye-conjugated secondary antibodies (IRDye®, LI-COR Biosciences, Lincoln, NE, USA), enabling simultaneous detection of target proteins and the housekeeping protein β-actin on the same membrane without stripping or re-probing. Secondary antibodies were applied for 1 h at room temperature in the dark. Immunoblots were imaged using a ChemiDoc system (Bio-Rad Laboratories, Hercules, CA, USA) operated with Image Lab software (version 6.1) (Bio-Rad Laboratories, Hercules, CA, USA) in multichannel mode, with IRDye 680 and IRDye 800 detected in separate infrared channels. Full, uncropped blots were acquired for each channel. For quantitative analysis, individual grayscale images corresponding to each channel were exported, and band intensities were quantified using ImageJ software (version 1.54f) (National Institutes of Health, Bethesda, MD, USA). Target protein expression was normalized to β-actin to control for loading variability. A complete list of primary antibodies is provided in Supplementary Table S2.

### 2.7. Imaging

THP-1 macrophages in 6- wells plate with coverslips were treated with fatty acids (oleic acid (OA), palmitic acid (PA), linoleic acid (LA), high-fat diet (HFD), and good-fat diet (GFD)). Cover slips were then washed with PBS and stained with the following immunofluorescent stains: BODIPY 493/503 (Invitrogen, Thermo Fisher Scientific, Eugene, OR, USA) to stain neutral lipid droplets and intracellular fat accumulation, incubated for 15min then washed with PBS (Gibco, Thermo Fisher Scientific, Waltham, MA, USA), followed by phalloidin staining for f-actin filaments labeling, cytoskeletal structure, and cell morphology, and DAPI staining for clear identification of the nucleus (both from Invitrogen, Thermo Fisher Scientific, Eugene, OR, USA). Both stains were incubated for 20 min and washed with PBS. Cover slips were then mounted on microscopic slides for imaging.

Imaging was conducted using an inverted Zeiss LSM710 spectral confocal microscope (Carl Zeiss, Gottingen, Germany), paired with an EC Plan-Neofluar 40×/1.30 oil DIC M27 objective lens. The samples underwent excitation utilizing a dual-laser system comprising a 543 nm HeNe laser and the 405 nm line of an argon ion laser, ensuring optimal illumination for each specific fluorophore. Following excitation, the emission detection bandwidths were optimized and configured using the Zeiss ZEN 2010 control software (version 6.0) (Carl Zeiss Microscopy, Göttingen, Germany). This careful calibration was crucial for accurately capturing the fluorescence emissions, thereby allowing for precise and detailed visualization of the cellular structures and components under study. The filter sets used corresponded to BODIPY (green; Ex/Em 493/503 nm), phalloidin (red), and DAPI (blue) channels. Microscope settings were carefully calibrated to prevent photobleaching and ensure precise visualization of cellular structures.

### 2.8. Flow Cytometry

THP-1 cells were seeded in a 12-well plate, treated with different fatty acids overnight. On the next day, cells were isolated, washed with PBS (Gibco, Thermo Fisher Scientific, Waltham, MA, USA), and stained with the following antibodies for M1/M2 ratios: CD11b (clone, D12)-APC (Cat#340936; BD Biosciences, San Jose, CA, USA), CD163-PE (Cat# 556018; BD Biosciences, San Jose, CA, USA), and HLA-DR-FITC (Cat# PN IM1838U; Beckman Coulter, Brea, CA, USA). To confirm the specificity of staining, isotype-matched control antibodies and non-stain cells were included in the staining protocol. Flow cytometry gating was performed to define target subsets, as seen in Supplementary Figure S1. In brief, singlets were first identified by gating forward scatter-height (FSC-H) versus forward scatter-area (FSC-A) to exclude doublets. Subsequently, macrophage populations were gated based on forward scatter (FSC) and side scatter (SSC) properties. M1+ subsets were defined as having a positive expression of CD11b+HLADr+. M2+ subsets were defined as having CD11b+CD163+ expression, and the ratio is defined as M1/M2.

To assess the profile of isolated PBMC, the stored cells underwent two washing cycles with 1 mL of PBS each. Cells were stained using the same antibody panel described above, with the addition of CD14 (BD Pharmingen™ PE-Cy™7 Mouse Anti-Human CD14, Cat#560919; BD Biosciences, San Jose, CA, USA).

Data acquisition was conducted using a BD FACSCanto II flow cytometer, and subsequent analysis was performed with DIVA software (version V6.1.3, BD Pharmingen; Biosciences, San Jose, CA, USA) or FlowJo V10.1.0.0.

### 2.9. Statistical Analysis

Unless otherwise stated, all experiments were performed using a minimum of n = 3 biological replicates. Biological replicates refer to independent experimental samples prepared and analyzed separately. Technical replicates were performed within each biological replicate and were averaged prior to statistical analysis.

Statistical analysis was performed using GraphPad Prism software version 10.6.1 (La Jolla, CA, USA). Data are shown as the mean ± the standard error of the mean (SEM) unless otherwise indicated. Parametric data were analyzed by one-way ANOVA followed by Tukey’s post hoc multiple comparisons test. For all analyses, data from a minimum of n = 3 were used for statistical calculation. ns = none significant, * *p* value < 0.05, ** *p* < 0.001, *** *p* < 0.001, and **** *p* < 0.0001 compared to respective control.

## 3. Results

### 3.1. Palmitate-Enriched Fatty Acid Composition Reduces Intracellular Lipid Accumulation and Alters Macrophage Morphology in THP-1-Derived Macrophages

**Figure 1:**
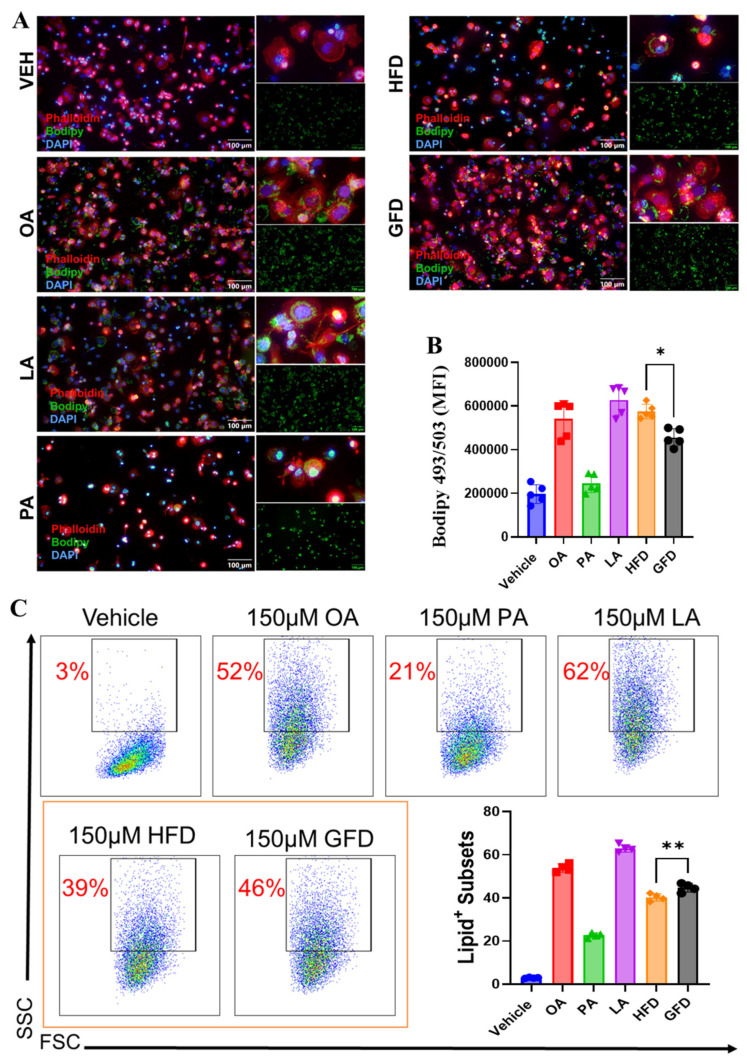
Palmitic acid-rich diet reduces intracellular lipid accumulation and alters macrophage morphology. THP-1-derived macrophages were stimulated for 24 h with defined lipid mixtures to mimic dietary fatty acid environments: a palmitic acid-enriched formulation (HFD; PA:OA:LA = 4:3:3) or an unsaturated fat-dominant mixture (GFD; PA:OA:LA = 2:4:4). (**A**) Representative confocal micrographs showing macrophages stained with phalloidin (red, F-actin cytoskeleton), BODIPY 493/503 (green, neutral lipids), and DAPI (blue, nuclei). Scale bars = 100 µm. HFD treatment markedly reduced intracellular lipid droplet accumulation compared to GFD, as seen by lower BODIPY signal intensity. (**B**) Quantification of intracellular lipid content based on BODIPY fluorescence intensity normalized to cell count. Data represent mean ± SEM from n = 3 independent experiments. (**C**) Flow cytometric analyses showing changes in cell complexity (side scatter, SSC) and size (forward scatter, FSC). Representative dot plots display increased granularity in HFD-treated macrophages, consistent with morphological activation. Along with quantification bar graph of granular (high-SSC) macrophage populations expressed as percentage of total cells. Data are presented as mean ± SEM (n = 3); * *p* < 0.05, ** *p* < 0.01 by one-way ANOVA with Tukey’s post hoc test.

To define how fatty acid composition influences macrophage lipid handling and cellular morphology, THP-1-derived macrophages were stimulated with defined lipid mixtures containing identical fatty acids but distinct ratios: a palmitate-enriched formulation (HFD; PA:OA:LA = 4:3:3) or an unsaturated fat-dominant mixture (GFD; PA:OA:LA = 2:4:4). Immunofluorescence staining with BODIPY revealed a significant reduction in intracellular neutral lipid accumulation in macrophages exposed to the palmitate-enriched mixture compared with the unsaturated fat-dominant condition (Figure 1A,B).

Notably, this reduction in lipid content was accompanied by pronounced morphological changes. Flow cytometric analysis of forward scatter (FSC) and side scatter (SSC) demonstrated increased cellular granularity in HFD-treated macrophages relative to GFD-treated cells (Figure 1C), consistent with cytoskeletal remodeling and altered cellular complexity. These phenotypic changes occurred despite reduced lipid accumulation, indicating that palmitate-enriched lipid environments induce structural and activation-associated remodeling that is uncoupled from intracellular lipid storage.

Together, these observations establish that fatty acid composition alone is sufficient to differentially regulate macrophage lipid handling and morphology in a controlled mechanistic system, providing a framework for subsequent analyses of inflammatory polarization and transcriptional programming.

### 3.2. Fatty Acid Composition Dictates Pro- and Anti-Inflammatory Polarization in Primary Human Macrophages

To determine whether fatty acid composition influences macrophage inflammatory polarization in a human context, primary human monocyte-derived macrophages were stimulated with defined lipid mixtures containing identical fatty acids but distinct ratios: a palmitate-enriched formulation (HFD; PA:OA:LA = 4:3:3) or an unsaturated fat-dominant formulation (GFD; PA:OA:LA = 2:4:4). Macrophage polarization was assessed by flow cytometric analysis of established surface markers associated with pro-inflammatory (M1-like) and anti-inflammatory (M2-like) phenotypes, using the gating strategy shown in Supplementary Figure S1.

**Figure 2:**
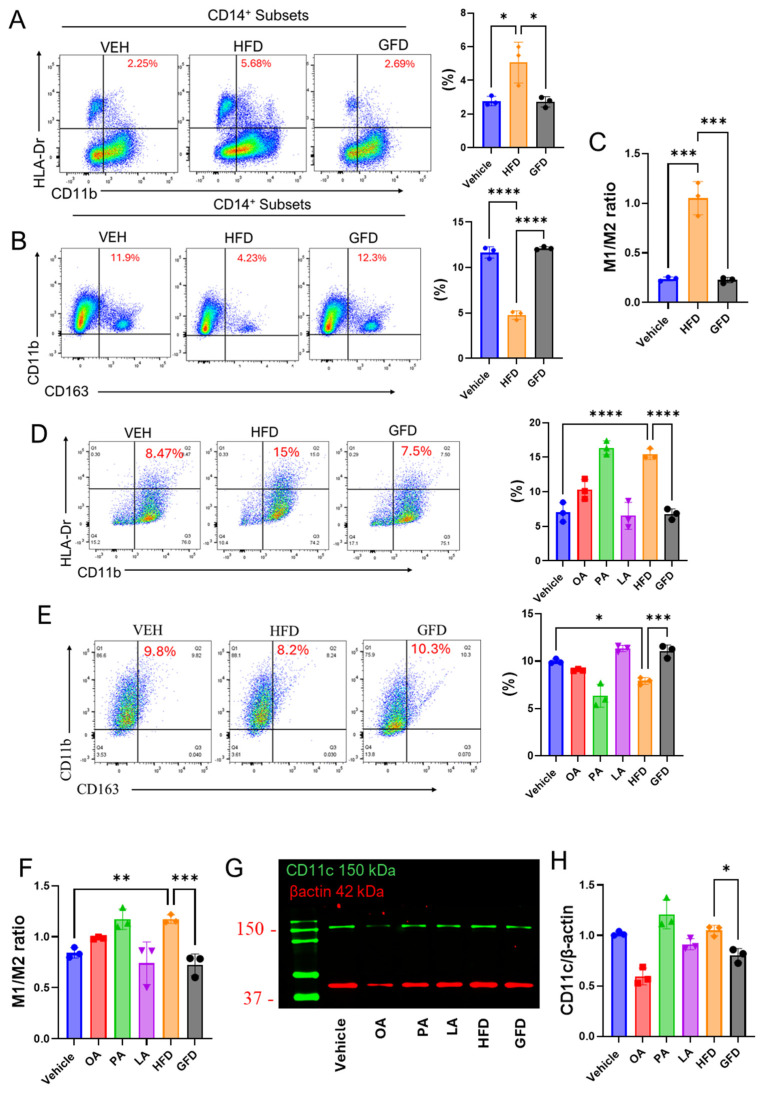
Fatty acid composition dictates pro- and anti-inflammatory macrophage polarization in human and THP-1-derived macrophages. To assess how defined fatty acid ratios regulate macrophage polarization, primary human monocyte-derived macrophages and THP-1-derived macrophages were stimulated for 24 h with vehicle (Control), a palmitate-enriched lipid mixture (HFD; PA:OA:LA = 4:3:3), or an unsaturated fat-dominant mixture (GFD; PA:OA:LA = 2:4:4). (**A**) Representative flow cytometry plots and quantification showing increased frequencies of CD14^+^CD11b^+^HLA-DR^+^ macrophages in primary human macrophages exposed to the palmitate-enriched mixture, consistent with M1-like polarization. (**B**) Representative plots and quantification demonstrating increased CD14^+^CD11b^+^CD163^+^ macrophages under unsaturated fat-dominant conditions, indicative of an M2-like phenotype in primary human macrophages. (**C**) Derived M1/M2 ratio in human macrophages, showing a significant shift toward pro-inflammatory polarization under palmitate-enriched conditions relative to GFD. To assess how distinct dietary fatty acid ratios modulate macrophage polarization in THP-1 model, THP-1-derived macrophages were stimulated in a similar manner. (**D**) Representative dot plots and quantification of CD11b^+^HLA-DR^+^ cells showing a significant increase in the M1-like population upon HFD treatment compared to control and GFD conditions. (**E**) Flow cytometric analysis and quantification of CD11b^+^CD163^+^ cells revealed that GFD treatment promoted an M2-like phenotype, whereas HFD exposure suppressed this anti-inflammatory subset. (**F**) Ratio of M1/M2 populations, demonstrating a pronounced shift toward M1 polarization in palmitate-enriched (HFD) conditions. (**G**,**H**) Representative immunoblot and densitometric quantification of CD11c (150 kDa) protein levels normalized to β-actin (42 kDa). GFD treatment reduced CD11c expression relative to HFD, further supporting an anti-inflammatory shift. Data are presented as mean ± SEM (n = 3 biological replicates, with each replicate representing an independent human donor.); * *p* < 0.05, ** *p* < 0.01, *** *p* < 0.001 and **** *p* < 0.0001 by one-way ANOVA with Tukey’s post hoc test.

Exposure to the palmitate-enriched lipid mixture significantly increased the proportion of CD14^+^CD11b^+^HLA-DR^+^ macrophages compared with both control and GFD-treated conditions, consistent with a pro-inflammatory polarization profile. In contrast, macrophages exposed to the unsaturated fat-dominant mixture displayed a significant increase in CD14^+^CD11b^+^CD163^+^ cells (Figure 2A,B), indicative of an anti-inflammatory phenotype. Accordingly, the M1/M2 ratio was markedly elevated under palmitate-enriched conditions relative to the unsaturated fat-dominant condition (Figure 2C), demonstrating that fatty acid composition alone is sufficient to bias human macrophage inflammatory fate.

It should be noted that macrophage polarization in this study is defined based on surface expression of HLA-DR and CD163 and therefore reflects relative M1-like and M2-like phenotypic bias rather than comprehensive functional or cytokine-based characterization. To establish mechanistic consistency across macrophage systems, these polarization patterns were next examined in THP-1-derived macrophages. Consistent with the human data, palmitate-enriched lipid exposure increased the frequency of CD11b^+^HLA-DR^+^ cells, whereas unsaturated fat dominance favored CD11b^+^CD163^+^ polarization (Figure 2D–F). Accordingly, these designations are used as operational markers of polarization trends rather than definitive inflammatory or anti-inflammatory macrophage states.

In addition, assessment of CD11c protein expression by multiplex Western blotting revealed a significant reduction in CD11c levels under GFD conditions, whereas no significant change was observed following HFD exposure (Figure 2G,H), further supporting an attenuation of pro-inflammatory programming in unsaturated fat-dominant environments.

Collectively, these findings demonstrate that fatty acid composition, rather than fat content per se, is a dominant determinant of macrophage polarization, with palmitate enrichment promoting pro-inflammatory programming and unsaturated fat dominance favoring anti-inflammatory phenotypes. Importantly, the concordance between primary human macrophages and THP-1-derived macrophages supports the translational relevance of the observed lipid ratio-dependent polarization.

### 3.3. Fatty Acid Composition Differentially Regulates PPAR Isoforms and Transcriptional Programs in Human and THP-1-Derived Macrophages

**Figure 3:**
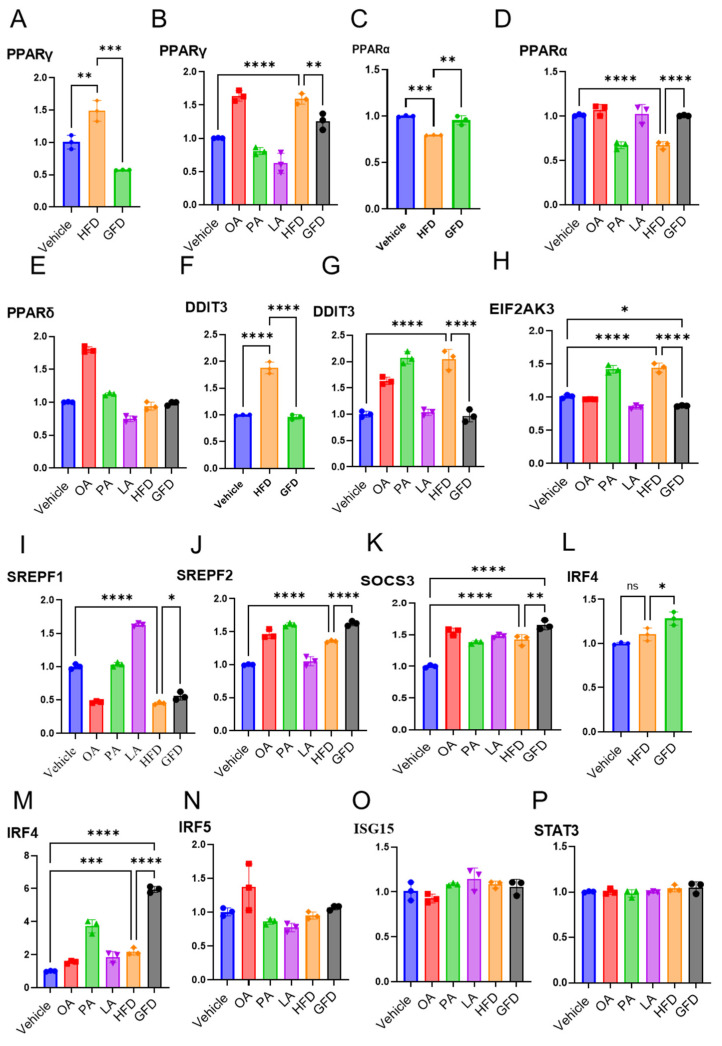
Differential regulation of PPARγ and PPARα defines the divergent effects of HFD and GFD on macrophage transcriptional programming. To delineate the molecular mechanisms driving lipid ratio-dependent macrophage polarization, primary human monocyte-derived macrophages and THP-1-derived macrophages were stimulated with either palmitate-enriched HFD (PA:OA:LA = 4:3:3) or unsaturated fat-dominant GFD (PA:OA:LA = 2:4:4) for 24 h, followed by transcriptional analysis of nuclear receptors and stress-responsive regulators. (**A**–**E**) Quantitative PCR analysis revealed selective induction of PPAR isoforms under HFD vs. GFD conditions. (**E**–**H**) The ER stress sensor DDIT3 (CHOP) and its upstream effector EIF2AK3 (PERK) were significantly upregulated in response to HFD, highlighting activation of the unfolded protein response under palmitate-rich conditions. (**I**,**J**) Expression of sterol regulatory element-binding transcription factors showed that SREBF1 was downregulated in both treatment groups, consistent with reduced lipogenesis, whereas SREBF2 was preferentially elevated in GFD-treated cells, suggesting enhanced cholesterol remodeling in response to unsaturated fatty acids. (**K**–**M**) The cytokine suppressor SOCS3 was increased under both lipid conditions, while IRF4 expression was modestly increased under unsaturated fat-dominant conditions, consistent with an associative rather than causative role in macrophage polarization. (**N**–**P**) Expression of IRF5, ISG15, and STAT3 remained unaltered, indicating selective transcriptional reprogramming rather than a global activation response. Data are presented as mean ± SEM (n = 3 biological replicates); * *p* < 0.05, ** *p* < 0.01, *** *p* < 0.001 and **** *p* < 0.0001 by one-way ANOVA with Tukey’s post hoc test.

To further define the molecular basis underlying lipid ratio-driven macrophage polarization, we examined the expression of PPAR isoforms and key transcriptional regulators in primary human monocyte-derived macrophages and THP-1-derived macrophages. Quantitative PCR analysis demonstrated that exposure to the palmitate-enriched lipid mixture (HFD) significantly increased PPARγ expression in primary human macrophages compared with control, whereas no significant induction was observed under GFD conditions (Figure 3A). A comparable pattern was observed in THP-1-derived macrophages, where HFD similarly induced PPARγ expression relative to control and GFD conditions (Figure 3B).

In contrast, PPARα expression displayed an opposing regulation. In primary human macrophages, GFD significantly increased PPARα expression, whereas palmitate enrichment suppressed its expression (Figure 3C). This reciprocal regulation was reproduced in THP-1-derived macrophages, confirming a conserved lipid ratio-dependent shift between PPARγ and PPARα-associated transcriptional programs across macrophage systems (Figure 3D). By comparison, PPARδ expression remained unchanged across lipid conditions (Figure 3E), indicating selective rather than global modulation of PPAR family members.

We next examined stress and metabolism-associated transcriptional regulators. The expression of the ER-stress effector DDIT3 (CHOP) was significantly increased following palmitate-enriched lipid exposure compared with both control and GFD conditions (Figure 3F,G). Consistently, expression of the ER-stress sensor EIF2AK3 (PERK) was also found to be elevated under palmitate-enriched conditions (Figure 3H), supporting activation of an unfolded protein response in palmitate-rich lipid environments.

Analysis of sterol regulatory factors revealed that SREBF1 expression was modestly reduced across lipid treatments (Figure 3I), whereas SREBF2 expression was preferentially increased under unsaturated fat-dominant conditions (Figure 3J), suggesting differential regulation of lipid metabolic pathways depending on fatty acid composition. Expression of SOCS3 showed a mild upward trend across lipid conditions (Figure 3K), consistent with a feedback response rather than a dominant regulatory shift.

Finally, we assessed transcription factors associated with macrophage inflammatory polarization. In primary human macrophages, IRF4 expression showed a modest increase under unsaturated fat-dominant conditions, suggesting a supportive transcriptional association rather than a dominant driver of anti-inflammatory programming (Figure 3L). In contrast, expression of IRF5, ISG15, and STAT3 in THP-1-derived macrophages did not differ significantly across lipid conditions (Figure 3M–P), indicating selective transcriptional reprogramming rather than a broad activation of inflammatory signaling pathways.

Collectively, these data demonstrate that fatty acid composition drives a coordinated transcriptional response across human and THP-1-derived macrophages, characterized by PPARγ induction and ER-stress activation under palmitate-enriched conditions, and PPARα- and IRF4-associated anti-inflammatory programming under unsaturated fat-dominant conditions. This conserved lipid ratio-dependent gene signature provides a mechanistic framework linking fatty acid composition to macrophage inflammatory fate and establishes the rationale for subsequent functional modulation of PPARγ signaling.

### 3.4. PPARγ Activity Is Necessary and Sufficient for Lipid Ratio-Dependent Macrophage Polarization

**Figure 4:**
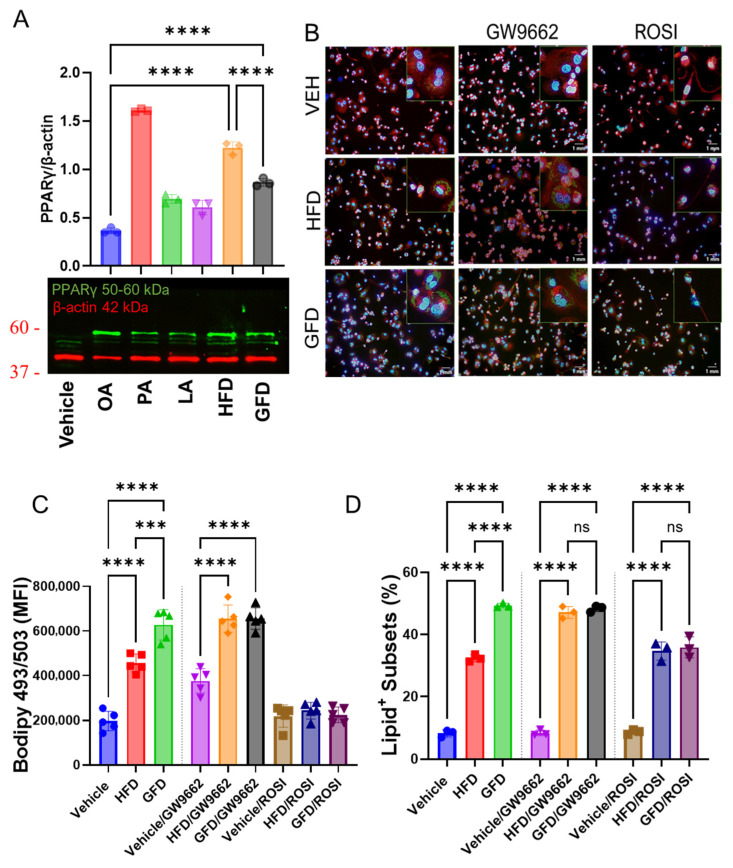
PPARγ activity is necessary and sufficient for lipid ratio-dependent macrophage polarization. To validate PPARγ expression at the protein level we conducted the following: (**A**) Representative immunoblot and densitometric quantification of PPARγ (50–60 kDa) protein levels normalized to β-actin (42 kDa). To functionally validate the role of PPARγ in mediating lipid ratio-driven macrophage polarization, THP-1-derived macrophages were treated with either the PPARγ antagonist GW9662 or the PPARγ agonist rosiglitazone under palmitate-enriched (HFD; PA:OA:LA = 4:3:3) or unsaturated fat-dominant (GFD; PA:OA:LA = 2:4:4) conditions for 24 h. (**B**) Representative confocal micrographs showing F-actin cytoskeleton (phalloidin, red), lipid droplets (BODIPY 493/503, green), and nuclei (DAPI, blue). HFD-treated macrophages exhibited reduced lipid accumulation and increased cell granularity compared to GFD, whereas pharmacologic inhibition of PPARγ with GW9662 restored intracellular lipid deposition and a rounded morphology under HFD conditions. Conversely, PPARγ activation with rosiglitazone in GFD-treated cells markedly reduced lipid content and promoted an elongated, stress-associated morphology. Insets highlight characteristic morphological features. Scale bars = 1 mm. (**C**,**D**) Quantitative analysis of lipid accumulation (BODIPY intensity per cell) and morphological parameters (granularity and circularity) confirmed that PPARγ inhibition reverses, while PPARγ activation enhances, lipid ratio-dependent phenotypic remodeling. Data represent mean ± SEM from three independent experiments; *** *p* < 0.001 and **** *p* < 0.0001 by one-way ANOVA with Tukey’s post hoc test.

To validate the transcriptional changes observed in Figure 3, we first examined PPARγ protein expression across individual fatty acids and lipid mixtures. Western blot analysis demonstrated that PPARγ protein levels were markedly elevated under palmitate-enriched (HFD) conditions, whereas GFD treatment resulted in lower expression, consistent with the mRNA trends (Figure 4A). These findings confirm that palmitate-rich lipid environments preferentially induce PPARγ at both the transcriptional and protein levels. To directly test whether PPARγ is required for fatty acid-driven macrophage phenotypes, we performed pharmacological loss- and gain-of-function experiments using the PPARγ antagonist GW9662 and the agonist rosiglitazone under both palmitate-enriched (HFD) and unsaturated fat-dominant (GFD) conditions. BODIPY-based imaging revealed that inhibition of PPARγ with GW9662 under HFD conditions increased intracellular lipid accumulation and partially restored a rounded cellular morphology, accompanied by reduced granularity, compared with vehicle-treated HFD cells. In contrast, pharmacological activation of PPARγ with rosiglitazone induced pronounced morphological remodeling and the reorganization of intracellular lipid droplets, producing BODIPY patterns consistent with altered droplet packing rather than a true reduction in total lipid content (Figure 4B–D).

Modulating PPARγ activity altered macrophage lipid handling and morphology irrespective of fatty acid composition, indicating that PPARγ activation is a dominant determinant of the observed phenotypes. Together, these findings demonstrate that PPARγ activity is both necessary and sufficient to mediate fatty acid-dependent macrophage remodeling and can override lipid composition when pharmacologically manipulated.

**Figure 5:**
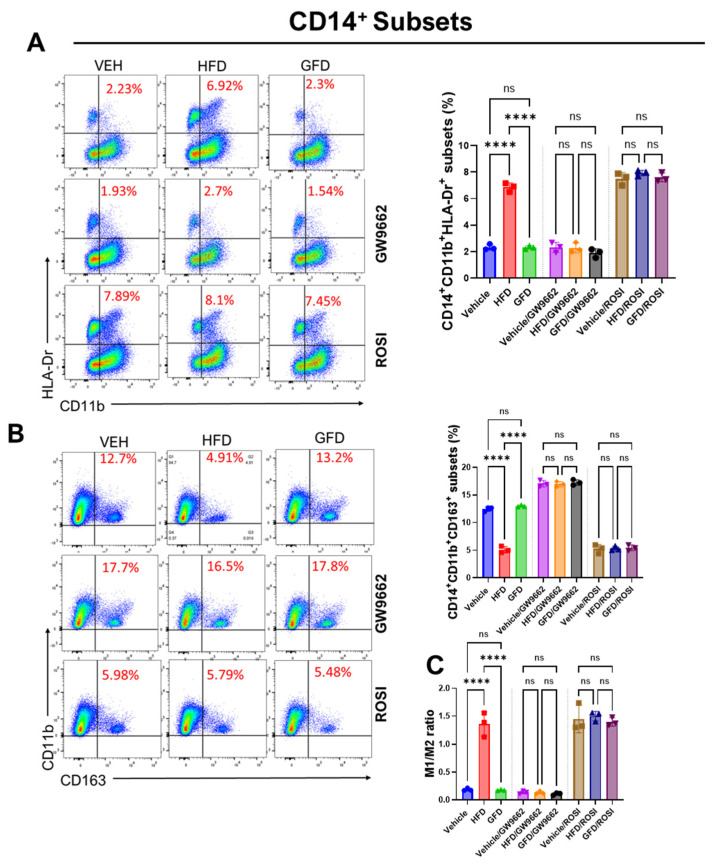
PPARγ modulation alters lipid-ratio-driven macrophage polarization in primary human monocyte-derived macrophages. Primary human macrophages isolated from PBMCs were treated with a palmitate-enriched lipid mixture (HFD; PA: OA:LA = 4:3:3) or an unsaturated fat-dominant mixture (GFD; PA:OA:LA = 2:4:4) for 24 h in the presence or absence of the PPARγ antagonist GW9662 or the PPARγ agonist rosiglitazone. (**A**,**B**) Representative flow cytometry plots showing CD14+CD11b^+^HLA-DR^+^ (M1-like) and CD14+CD11b^+^CD163^+^ (M2-like) subsets under each condition. (**C**) Quantification of M1, M2, and M1/M2 ratios. Data represent mean ± SEM (n = 3 biological replicates, with each replicate representing an independent human donor.); **** *p* < 0.0001 by one-way ANOVA with Tukey’s post hoc test.

We next assessed macrophage polarization by flow cytometry in primary human monocyte-derived macrophages to determine whether PPARγ activity functionally regulates lipid ratio-dependent inflammatory programming. Pharmacological inhibition of PPARγ with GW9662 significantly attenuated the HFD-induced increase in CD14^+^CD11b^+^HLA-DR^+^ macrophages, thereby blunting the pro-inflammatory M1 bias and partially restoring the M1/M2 balance. In contrast, activation of PPARγ with rosiglitazone under unsaturated fat-dominant (GFD) conditions significantly increased the proportion of CD14^+^CD11b^+^HLA-DR^+^ cells while reducing CD14^+^CD11b^+^CD163^+^ macrophages, resulting in an elevated M1/M2 ratio (Figure 5A–C). These findings indicate that PPARγ activity is sufficient to override the anti-inflammatory programming induced by unsaturated fatty acid-dominant environments in human macrophages.

Mechanistic consistency was subsequently examined in THP-1-derived macrophages, where comparable PPARγ-dependent effects were observed. In this model, GW9662 similarly attenuated HFD-driven M1 polarization, whereas rosiglitazone reversed the GFD-associated M2 phenotype, supporting a conserved role for PPARγ signaling in lipid ratio-dependent macrophage polarization (Supplementary Figure S2).

Together, these experiments demonstrate that PPARγ is both necessary and sufficient to dictate lipid ratio-dependent macrophage morphology, lipid metabolism, and polarization. Specifically, PPARγ inhibition alleviates palmitate-induced stress remodeling and M1 skewing, whereas its activation disrupts the protective programming elicited by unsaturated fatty acids. These results establish PPARγ as a central molecular switch linking dietary lipid composition to macrophage fate.

## 4. Discussion

In this study, we demonstrate that the relative composition of dietary fatty acids rather than their absolute presence profoundly influences macrophage lipid metabolism, morphology, and polarization. Palmitate-enriched mixtures (HFD) promoted a stress-associated phenotype characterized by reduced lipid accumulation, increased granularity, induction of PPARγ, and upregulation of ER stress mediators (EIF2AK3, DDIT3), leading to a predominance of M1-like CD11b^+^HLA-DR^+^ macrophages. By contrast, unsaturated fat-dominant mixtures (GFD) favored rounded macrophages with abundant lipid droplets, induction of PPARα, IRF4, and SREBF2, and a bias toward an M2-like CD11b^+^CD163^+^ phenotype. Functional studies further established PPARγ as a central regulator, as pharmacologic inhibition attenuated HFD-induced M1 polarization, whereas agonist activation disrupted the protective program elicited by GFD. Together, these results highlight a lipid ratio-sensing role for PPARγ, situating it as a molecular switch that links dietary lipid quality to macrophage fate.

Our findings are consistent with prior evidence that PPARγ acts as a lipid-activated transcription factor coupling metabolic inputs to macrophage differentiation and effector functions. Previous studies have reported that palmitate activates inflammatory and ER stress pathways in macrophages, in part through PERK-CHOP signaling, thereby exacerbating metabolic dysfunction. Here, we extend this paradigm by showing that palmitate enrichment is accompanied by PPARγ induction, which, rather than exerting its classical anti-inflammatory role, is co-opted into a stress-associated program. Nonetheless, emerging evidence indicates that post-translational modifications of PPARγ, together with context-dependent co-factor recruitment, can fundamentally alter its transcriptional output, shifting PPARγ signaling from anti-inflammatory programs toward stress-associated and maladaptive lipid-handling pathways [6,13,14].

Traditionally, PPARγ has been regarded as an anti-inflammatory nuclear receptor; however, accumulating evidence, including our own findings, indicates that this view is overly simplistic. PPARγ activity is highly context-dependent and is strongly shaped by the lipid environment in which it is engaged [13]. In palmitate-enriched settings, PPARγ induction coincides with ER-stress signaling, reduced lipid handling, and a shift toward an M1-like pro-inflammatory phenotype. This aligns with emerging reports showing that post-translational modifications, co-factor availability, and lipid-derived ligands can reprogram PPARγ toward maladaptive inflammatory functions [14]. In sharp contrast, unsaturated fat-dominant environments promote PPARγ-independent signaling via PPARα and IRF4, supporting alternative activation and metabolic remodeling. These observations underscore that PPARγ does not exert a fixed anti-inflammatory effect; rather, its function depends critically on the quality of available lipids, providing a mechanistic basis for the differential macrophage responses observed in our study [6].

Conversely, our data indicate that unsaturated fatty acids bias macrophages toward PPARα- and IRF4-driven programs, consistent with the well-documented anti-inflammatory actions of oleate and linoleate [15,16]. Importantly, the fatty acid mixtures used in this study induce modulatory, context-dependent shifts in polarization markers rather than the maximal canonical M1 or M2 activation typically achieved with cytokine stimuli such as LPS/IFNγ or IL-4/IL-13.

While IRF4 has been implicated in alternative macrophage activation, its modest induction in primary human macrophages in the present study suggests a supportive or permissive role rather than a primary mechanistic driver. Interestingly, it has been reported that IRF4 deficiency abrogates M2 polarization and worsens metabolic inflammation [17]. The upregulation of SREBF2, particularly under GFD, suggests adaptive cholesterol remodeling, in agreement with recent reports identifying SREBP2 as a regulator of cholesterol biosynthesis and macrophage immune function [18]. Collectively, these results point to a coordinated transcriptional network wherein PPARγ and ER stress pathways dominate in palmitate-rich contexts, while PPARα, IRF4, and SREBF2 sustain protective programming under unsaturated fat-rich conditions.

Our functional experiments reinforce the necessity and sufficiency of PPARγ activity in determining lipid ratio-dependent polarization. Antagonism with GW9662 alleviated HFD-induced M1 skewing and restored lipid accumulation, while activation with rosiglitazone in GFD-exposed macrophages overrode the protective M2 phenotype. While rosiglitazone and other thiazolidinediones are often described as anti-inflammatory in vivo [19], our results highlight the importance of contextual lipid environments: in a palmitate-enriched milieu, PPARγ activation cooperates with stress pathways to drive inflammation, whereas in a balanced or unsaturated-rich milieu, it may facilitate resolution. These findings underscore the importance of dietary lipid quality in shaping the outcome of PPARγ-targeted interventions.

Future studies should validate rosiglitazone’s actions using additional PPARγ agonists such as pioglitazone or troglitazone, as well as by assessing established PPARγ target genes, to further strengthen the mechanistic interpretation of PPARγ-dependent lipid remodeling.

### Limitations

Several limitations of this study should be acknowledged. First, the present study utilized a 24 h exposure model to capture early lipid-sensing and transcriptional events. Accordingly, the present model is not intended to recapitulate chronic dietary lipid exposure in vivo, which is more appropriately addressed using animal models or tissue-resident macrophages. These rapid responses, including PPAR activation, ER-stress signaling, oxidative-stress pathways, and early M1/M2 polarization, are well supported by our previous work using acute palmitate, LPS, SCFA, and EPA models. While this timeframe is appropriate for dissecting early mechanistic programming, it does not model chronic or unresolved inflammation. Future studies using longer exposures (48–72 h or more) or primary tissue-resident macrophages will be required to examine sustained inflammatory states.

Second, our study employed THP-1-derived macrophages and defined lipid mixtures, which provide mechanistic clarity but may not fully recapitulate tissue-resident macrophage diversity or complex post-prandial lipoprotein environments. Third, we relied on pharmacologic modulators of PPARγ; while GW9662 and rosiglitazone are widely used, genetic approaches (e.g., CRISPR/Cas9 or siRNA knockdown) would provide stronger evidence of causality. In addition, while our data establish a functional requirement for PPARγ activity in palmitate-induced stress and inflammatory phenotypes, we did not directly assess PPARγ chromatin occupancy or transcriptional binding to ER-stress or pro-inflammatory gene loci; such analyses (e.g., ChIP-based approaches) will be required to define direct regulatory mechanisms. Fourth, our transcriptional analysis focused on a targeted panel of regulators; genome-wide transcriptomic and epigenomic profiling would further illuminate the broader network of PPARγ-dependent gene programs. Finally, macrophage polarization was assessed using a limited surface marker panel, and while informative, this approach does not capture the full functional or transcriptional heterogeneity of macrophage activation states in vivo. Our reliance on the M1/M2 framework, while useful for interpretation, simplifies the full continuum of macrophage states observed [20].

## 5. Conclusions

In conclusion, our study identifies PPARγ as a critical molecular switch that interprets dietary fatty acid ratios to direct macrophage lipid handling, morphology, and inflammatory polarization. Palmitate-rich environments co-opt PPARγ into a stress-associated program, whereas unsaturated fat-rich environments favor PPARα-associated transcriptional remodeling accompanied by modest IRF4 induction, collectively supporting an anti-inflammatory macrophage bias. These findings not only advance our understanding of how dietary lipid quality modulates innate immunity but also highlight the need for context-specific strategies when targeting PPARγ pharmacologically.

## Data Availability

All data supporting the findings of this study are presented within the manuscript. No additional publicly archived datasets were generated or analyzed during the current study. The corresponding author, Fatema Al-Rashed, will gladly provide further information or raw data upon reasonable request and with appropriate justification.

## Supplementary Materials

The following supporting information can be downloaded at https://www.mdpi.com/article/10.3390/cells15030308/s1: Figure S1: Flow cytometry gating strategy for macrophage polarization analysis in primary human monocyte-derived macrophages and THP-1–derived macrophages; Figure S2: PPARγ modulation alters lipid-ratio–driven macrophage polarization in, THP-1 derived macrophages; Table S1: Primers used for gene expression analysis in this study; Table S2: Primary and secondary antibodies employed in Western blotting.

## Abbreviations

The following abbreviations are used in this manuscript:

Abbreviation	Definition
ACSL1	Acyl-CoA Synthetase Long Chain Family Member 1
ANOVA	Analysis of Variance
BODIPY	Boron-Dipyrromethene
BSA	Bovine Serum Albumin
CVD	Cardiovascular Disease
DAPI	4′,6-Diamidino-2-Phenylindole
DDIT3	DNA Damage Inducible Transcript 3 (CHOP)
EIF2AK3	Eukaryotic Translation Initiation Factor 2 Alpha Kinase 3 (PERK)
ER	Endoplasmic Reticulum
FA	Fatty Acid
FSC	Forward Scatter
GFD	Good-Fat Diet
HFD	High-Fat Diet
HLA-DR	Human Leukocyte Antigen–DR Isotype
IF	Immunofluorescence
IRDye	Infrared Dye
IRF	Interferon Regulatory Factor
ISG15	Interferon-Stimulated Gene 15
LA	Linoleic Acid
LFD	Low-Fat Diet
M1	Classically Activated (Pro-Inflammatory) Macrophage
M2	Alternatively Activated (Anti-Inflammatory) Macrophage
mRNA	Messenger Ribonucleic Acid
OA	Oleic Acid
PA	Palmitic Acid
PBMC	Peripheral Blood Mononuclear Cell
PBS	Phosphate-Buffered Saline
PCR	Polymerase Chain Reaction
PPAR	Peroxisome Proliferator-Activated Receptor
PPARα	Peroxisome Proliferator-Activated Receptor Alpha
PPARδ	Peroxisome Proliferator-Activated Receptor Delta
PPARγ	Peroxisome Proliferator-Activated Receptor Gamma
qPCR	Quantitative Polymerase Chain Reaction
RNA	Ribonucleic Acid
RPMI	Roswell Park Memorial Institute Medium
SREBF	Sterol Regulatory Element-Binding Transcription Factor
SSC	Side Scatter
STAT3	Signal Transducer and Activator of Transcription 3
THP-1	Human Monocytic Leukemia Cell Line
TLR	Toll-Like Receptor
UPR	Unfolded Protein Response
