# Supplementary material for: Fatty Acid Composition, at Equivalent Lipid Exposure, Dictates Human Macrophage Polarization via PPARγ Signaling
Source: Cells. 2026 Feb 6;15(3):308. doi: 10.3390/cells15030308 (PMC12897183; doi:10.3390/cells15030308)
Supplement: Supplementary file 1 [file cells-15-00308-s001.zip › Supplementary Figure S1.pdf]

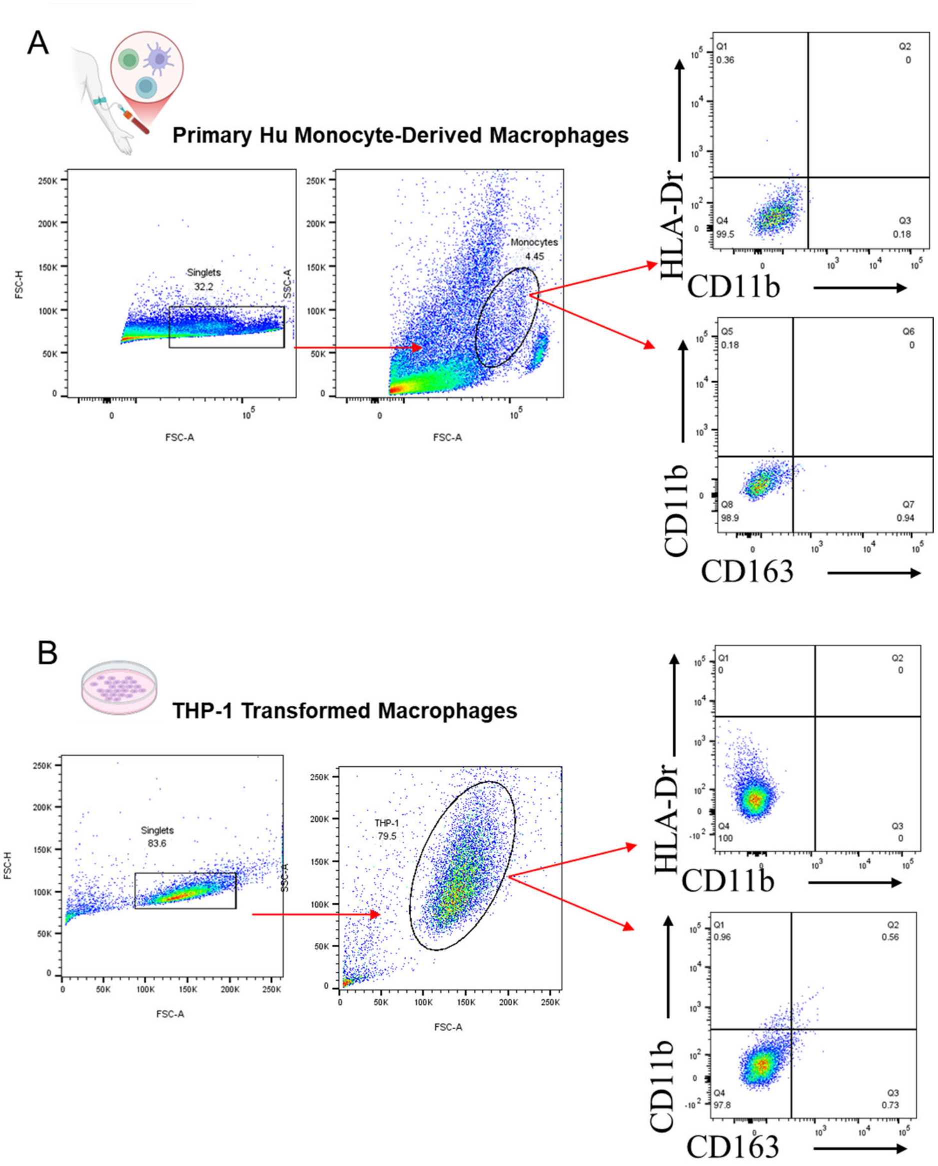

Supplementary Figure S1. Flow cytometry gating strategy for macrophage polarization analysis in primary human monocyte-derived macrophages and THP-1–derived macrophages. (A) For primary human monocyte-derived macrophages, singlets were first identified by forward scatter height versus forward scatter area (FSC-H vs. FSC-A) to exclude doublets, followed by gating based on forward scatter (FSC) and side scatter (SSC) properties to define the monocyte/macrophage population. From this parent gate, surface marker expression was assessed using quadrant analysis to identify CD11b^{+}HLA-DR^{+} (M1-like) and CD11b^{+}CD163^{+} (M2-like) subsets, as indicated. (B) For THP-1–derived macrophages, an identical gating strategy was applied, including singlet discrimination (FSC-H vs. FSC-A), FSC/SSC-based macrophage gating, and subsequent identification of CD11b^{+}HLA-DR^{+} and CD11b^{+}CD163^{+} populations. Percentages shown in each quadrant represent the proportion of cells within the respective parent gate. This gating strategy was applied uniformly across all experimental conditions and was used for quantification of M1-like, M2-like, and M1/M2 ratios presented in the main figures.
