# Supplementary material for: Fatty Acid Composition, at Equivalent Lipid Exposure, Dictates Human Macrophage Polarization via PPARγ Signaling
Source: Cells. 2026 Feb 6;15(3):308. doi: 10.3390/cells15030308 (PMC12897183; doi:10.3390/cells15030308)
Supplement: Supplementary file 1 [file cells-15-00308-s001.zip › Supplementary Figure S2.pdf]

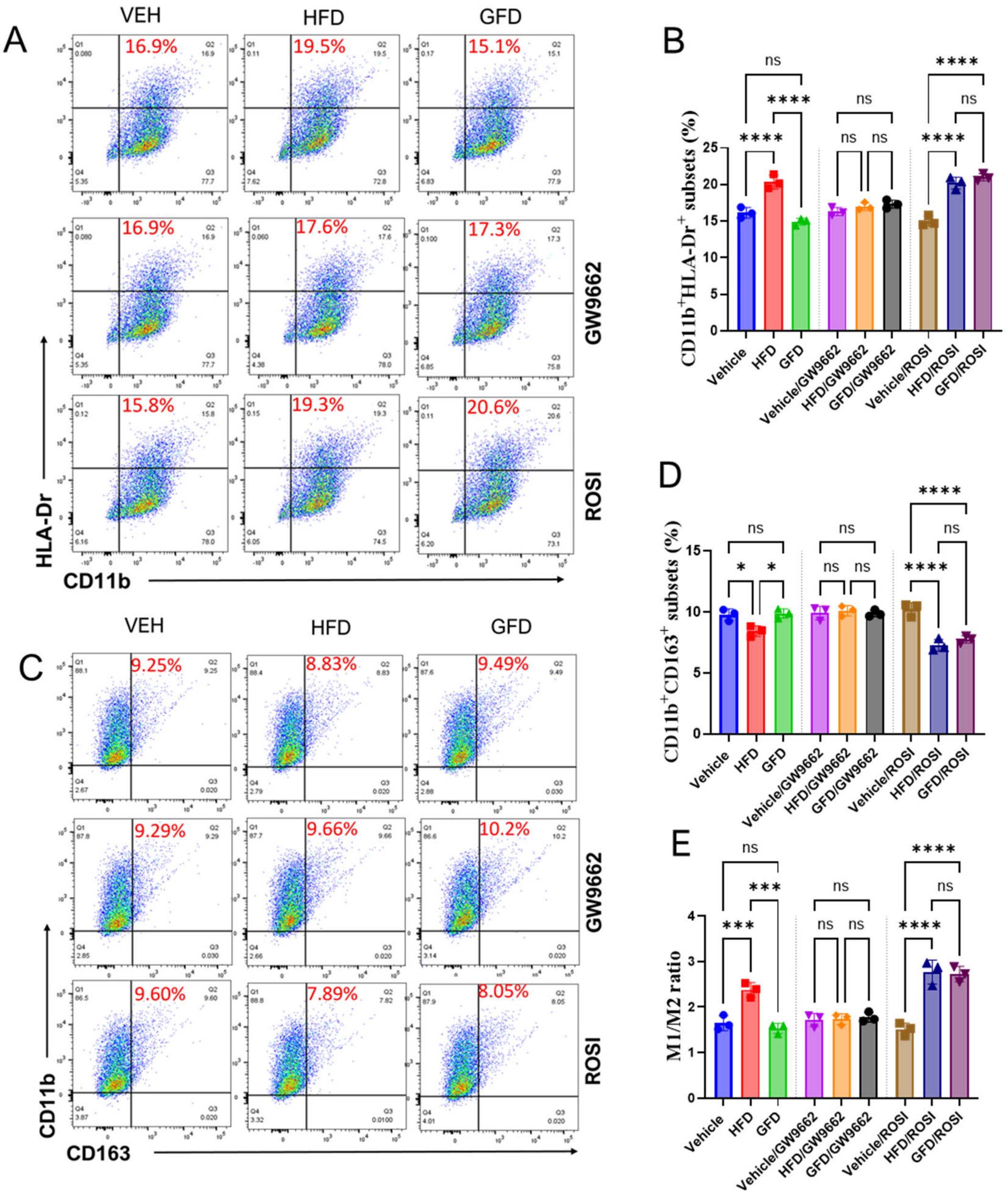

Supplementary Figure S2. PPARγ modulation alters lipid-ratio–driven macrophage polarization in , THP-1 derived macrophages . To determine whether PPARγ activity directly regulates macrophage polarization in response to distinct lipid environments, THP-1 derived macrophages were treated with either the PPARγ antagonist GW9662 or the PPARγ agonist rosiglitazone under HFD and GFD conditions. (A–B) Representative flow cytometry plots showing expression of $CD11b^{+}HLA\text{-}DR^{+}$ (M1) and $CD11b^{+}CD163^{+}$ (M2) macrophages under the indicated treatments. HFD induced a significant expansion of $CD11b^{+}HLA\text{-}DR^{+}$ cells compared to control, which was markedly attenuated by GW9662 treatment. (C-D) Conversely, rosiglitazone treatment under GFD conditions increased $CD11b^{+}HLA\text{-}DR^{+}$ macrophages while reducing $CD11b^{+}CD163^{+}$ populations, resulting in a higher M1/M2 ratio. (E) Quantitative analysis of M1, M2, and M1/M2 ratios con-firmed that inhibition of PPARγ blunted the pro-inflammatory response induced by HFD, whereas forced activation of PPARγ reversed the anti-inflammatory bias of GFD. Data represent mean ± SEM from n = 3 independent experiments; $p < 0.05$, $p < 0.01$, $p < 0.001$ by one-way ANOVA with Tukey's post-hoc test.
