# Supplementary material for: Fatty Acid Composition, at Equivalent Lipid Exposure, Dictates Human Macrophage Polarization via PPARγ Signaling
Source: Cells. 2026 Feb 6;15(3):308. doi: 10.3390/cells15030308 (PMC12897183; doi:10.3390/cells15030308)
Supplement: Supplementary file 1 [file cells-15-00308-s001.zip › Supplementary Table S1.pdf]

Supplementary Table S1. Primers used for gene expression analysis in this study

Oligonucleotides	Source	Catalog
Primers for: DDIT3	Thermofisher	Cat#4331182; Assay ID: Hs00358796_g1
Primers for: EIF2AK3	Thermofisher	Cat#4331182; Assay ID: Hs00984003_m1
Primers for: GAPDH	Thermofisher	Cat#4331182; Assay ID: Hs03929097_g1
Primers for: IRF4	Thermofisher	Cat#4331182; Assay ID: Hs00180031_m1
Primers for: IRF5	Thermofisher	Cat#4331182; Assay ID: Hs00158114_m1
Primers for: ISG15	Thermofisher	Cat#4331182; Assay ID: Hs01921425_s1
Primers for: PPAR ALPHA	Thermofisher	Cat#4331182; Assay ID: Hs00947536_m1
Primers for: PPAR DELTA	Thermofisher	Cat#4331182; Assay ID: Hs04187066_g1
Primers for: PPAR GAMMA	Thermofisher	Cat#4331182; Assay ID: Hs01115513_m1
Primers for: SOCS3	Thermofisher	Cat#4331182; Assay ID: Hs02330328_s1
Primers for: SREBF1 FAM	Thermofisher	Cat#4331182; Assay ID: Hs02561944_s1
Primers for: SREBF2	Thermofisher	Cat#4331182; Assay ID: Hs01081784_m1
Primers for: STAT3	Thermofisher	Cat#4331182; Assay ID: Hs00374280_m1
