# Supplementary material for: Fatty Acid Composition, at Equivalent Lipid Exposure, Dictates Human Macrophage Polarization via PPARγ Signaling
Source: Cells. 2026 Feb 6;15(3):308. doi: 10.3390/cells15030308 (PMC12897183; doi:10.3390/cells15030308)
Supplement: Supplementary file 1 [file cells-15-00308-s001.zip › Supplementary Table S2.pdf]

Supplementary Table S2. Primary and secondary antibodies employed in Western blotting

Antibodies	Source	Catalog
Beta Actin (8H10D10) Mouse mAb	Cell Signaling	3700T
CD11C (D3V1E) (XP) ® Rabbit mAb	Cell Signaling	45581S
IRDye® 680RD	Abcam	ab216777
IRDye® 800CW	Abcam	ab216772
